# Bacterial and Fungal Pathogens in Chronic Suppurative Otitis Media and Otitis Externa With Persistent Otorrhea: A Cross-Sectional Study in a Low- to Middle-Income Country (Pakistan)

**DOI:** 10.7759/cureus.84147

**Published:** 2025-05-15

**Authors:** Shanila Feroz, Ejaz Ur Rehman, Muhammad Saad Raza, Mehrozia Nuzhat, Asma Noreen, Marion Samson

**Affiliations:** 1 Ear, Nose, and Throat, United Medical and Dental College, Karachi, PAK; 2 Pediatrics, United Medical and Dental College, Karachi, PAK; 3 Pediatrics, Jinnah Medical and Dental College, Karachi, PAK

**Keywords:** bacterial pathogens, chronic suppurative otitis media, cross sectional study, fungal pathogens, otitis externa, persistent otorrhea

## Abstract

Objective: This study aimed to determine the prevalence and distribution of bacterial and fungal pathogens in cases of chronic suppurative otitis media (CSOM) and otitis externa presenting with persistent otorrhea and to compare associated pathogen profiles between these two conditions in a low-middle-income country setting (Pakistan).

Design, setting, and duration: This cross-sectional study was conducted at Creek General Hospital and United Hospital in Karachi, Pakistan, over a three-month period from 1 December 2024 till 28 February 2025.

Methods: A total of 384 patients aged 11-65 years diagnosed with CSOM or otitis externa and exhibiting persistent otorrhea were recruited through convenient sampling. Demographic and microbiological data, including pathogen identification and characterization, were collected using a structured evaluation form. Data analysis employed Statistical Product and Service Solutions (SPSS, version 27; IBM SPSS Statistics for Windows, Armonk, NY), with the chi-square test to assess variable associations and significant variations between the groups (p < 0.05 considered significant).

Primary outcome: To identify the frequency of common pathogen types isolated from patients with CSOM and otitis externa.

Secondary outcome: The comparative distribution of bacterial and fungal pathogens isolated from patients diagnosed with CSOM and otitis externa presenting with persistent otorrhea.

Results: Of 384 participants, 50.8% were male, and 49.2% were female, predominantly aged 21-30 years and from urban areas. CSOM was the most prevalent diagnosis (46.6%), followed by otitis externa (42.7%), with co-infections in 10.7%. Bacterial pathogens (50%) were more common than fungal (28.6%) or mixed infections (21.4%). The most frequently identified bacterial pathogen was Pseudomonas aeruginosa (34.6%), while Aspergillus spp. (49.7%) predominated among fungi. Pathogen-type distribution showed no significant variation between CSOM and otitis externa (p > 0.05).

Conclusion: This study highlights the high prevalence of P. aeruginosa and Aspergillus spp. as the most common pathogens in CSOM and otitis externa in Karachi, Pakistan. The findings underscore the importance of pathogen-specific treatment protocols tailored to local microbial patterns. Given the region's environmental factors, such as poor sanitation and humidity, integrating these microbial profiles into local healthcare strategies is crucial for effective antimicrobial stewardship. There was no significant variation between pathogen distribution across CSOM and otitis externa (p > 0.05), indicating that the choice of pathogen was not strongly linked to clinical condition. While this study did not explore the links between pathogen type and clinical severity or treatment outcomes, these results provide valuable insights for guiding clinicians in optimizing treatment regimens. Ultimately, such strategies could improve patient outcomes and reduce the burden of these infections in similar low-resource settings.

## Introduction

Chronic suppurative otitis media (CSOM) and otitis externa are common ear infections that contribute significantly to global morbidity, with a notably higher burden in tropical and subtropical regions [1,2], particularly in low- and middle-income countries, where access to timely and effective treatment is limited. CSOM is a persistent infection of the middle ear characterized by recurrent ear discharge (otorrhea) through a perforated tympanic membrane [3], while otitis externa refers to inflammation of the external auditory canal, often accompanied by chronic otorrhea [2]. Although anatomically distinct, both conditions are frequently encountered in otologic practice, may coexist in clinical settings, and are often associated with persistent otorrhea, making comparative microbial analysis relevant. If left untreated, both infections can result in serious complications, including hearing loss, mastoiditis, and even intracranial spread [4-6]. These infections are commonly polymicrobial, involving a range of bacterial and fungal pathogens [7-12], with pathogen profiles varying depending on geographical location, access to healthcare, and environmental factors [13-16]. In regions such as Karachi, Pakistan, the warm, humid climate, coupled with poor sanitation and limited healthcare infrastructure, creates an ideal environment for the growth of both bacterial and fungal pathogens, complicating the management of these infections [16,17]. Despite the significant prevalence of CSOM and otitis externa in such areas, particularly with persistent otorrhea (ongoing aural discharge lasting more than two weeks despite standard therapy), there is a lack of detailed data regarding the microbial profiles associated with these conditions. Defining the local prevalence and distribution of microbial pathogens in these infections is essential for optimizing empiric treatment, curbing antimicrobial resistance, and minimizing complications. Therefore, we aimed to determine the prevalence and distribution of bacterial and fungal pathogens in patients diagnosed with CSOM and otitis externa presenting with persistent otorrhea in Karachi, Pakistan, and to compare pathogen profiles between the two conditions to evaluate any significant differences in microbial etiology. This study is expected to fill a critical gap in regional microbiological data and inform targeted, evidence-based antimicrobial therapy to improve clinical outcomes in resource-limited tropical settings.

## Materials and methods

Study design, setting, and duration

This cross-sectional study was conducted at Creek General Hospital and United Hospital, Karachi, Pakistan, over a three-month period from 1 December 2024 till 28 February 2025. Ethical approval was obtained from the Institutional Review Board of United Medical and Dental College, Karachi, Pakistan (UMDC/Ethics/2024/11/20/374), ensuring adherence to the ethical principles outlined in the Declaration of Helsinki.

Sample size, technique, and participants

An initial sample size of 126 patients was calculated using the OpenEpi (Centers for Disease Control and Prevention, Atlanta, GA) sample size calculator, based on a 9% average prevalence of CSOM and otitis externa in Pakistan. However, to improve the statistical power, enhance the representativeness of findings, and ensure the inclusion of all eligible cases presenting within the defined study period, the final sample was expanded to 384 patients. This increase was not based on a formal post-pilot recalculation but rather on pragmatic considerations related to feasibility and availability of cases during the recruitment window in two high-volume urban hospitals. The participants were recruited using convenience sampling. This approach was chosen due to its practicality and feasibility within the limited time frame and available resources. The study was conducted in high-volume urban hospitals where eligible patients frequently presented with persistent otorrhea, facilitating efficient recruitment and timely microbiological analysis. The final sample recruited was 384 to enhance the significance of the representation.

Inclusion and exclusion criteria

Patients aged 11-65 years, diagnosed with CSOM or otitis externa, exhibiting persistent otorrhea, and seeking treatment at Creek General Hospital or United Hospital were included in the study. Patients with a history of prior ear surgeries, those currently on antibiotic therapy, and individuals unwilling to provide informed consent or missing data were excluded.

Data collection

Data were collected using a self-administered evaluation form designed to capture the prevalence and characterization of bacterial and fungal pathogens associated with CSOM and otitis externa (Appendix Table 4). The form included sections, which consisted of categorical items such as demographic data, and microbiological data (identification and characterization of pathogens), which were filled out by clinicians based on patient interviews and microbiological results, rather than by patients themselves. A pilot study involving 20 patients was conducted to validate the study tool for data entry and ensure feasibility, although the pilot data were not included in the final analysis. While the tool was not formally validated through psychometric testing, its structured design and clinician-based administration ensured reliability and reproducibility within the study's clinical context. Data collectors and laboratory personnel were blinded to the patients' clinical diagnoses and any pre-existing assumptions regarding pathogen type to reduce detection and observer bias. All data were anonymized before entry and analysis. Participants provided informed consent prior to enrollment, and data collection occurred in person during outpatient department (OPD) visits.

Microbiological testing

Ear discharge samples were collected under aseptic conditions using sterile cotton swabs. The swabs were immediately placed into sterile transport media and transported to the microbiology laboratory within one hour of collection. Specimens were processed using standard microbiological protocols. Bacterial cultures were inoculated on blood agar, MacConkey agar, and chocolate agar and incubated at 35-37°C for 24-48 hours under aerobic conditions. Fungal cultures were performed using Sabouraud Dextrose Agar (SDA) and incubated at 25-28°C for up to seven days. Microorganisms were identified based on colony morphology, Gram staining, and standard biochemical tests. Fungal identification was aided by lactophenol cotton blue staining and colony characteristics. Mixed infections were identified based on the isolation of more than one organism (bacterial and/or fungal) from a single clinical specimen. Clinical suspicion alone was not used to define mixed infections; identification relied solely on laboratory results.

Statistical analysis

Data were manually entered into Excel (Microsoft® Corp., Redmond, WA) and analyzed using Statistical Product and Service Solutions (SPSS, version 27; IBM SPSS Statistics for Windows, Armonk, NY). Descriptive statistics summarized categorical variables as frequencies and percentages. A chi-square test was applied to assess relationships between variables, with a p-value ≤ 0.05 considered statistically significant. Independent variables included age, sex, residence, and diagnosis, while dependent variables focused on the prevalence and characterization of bacterial and fungal pathogens. The study adhered to strict ethical guidelines, ensuring participant confidentiality, with data accessible only to authorized researchers. Written informed consent was obtained from all participants, and the reporting followed the Strengthening the Reporting of Observational studies in Epidemiology (STROBE) guidelines to ensure comprehensive and standardized documentation.

## Results

Demographic characteristics

Of the 384 participants, 50.8% were males, and 49.2% were females. The majority of participants were between the ages of 21-30 years (31.3%), with most residing in urban areas (60.7%) (Table 1).

**Table 1:** Demographic data of the participants (N=384).

Variables	Frequency (n)	Percentage (%)
Sex		
Male	195	50.8%
Female	189	49.2%
Age		
11-20	38	9.9%
21-30	120	31.3%
31-40	90	23.4%
41-50	65	16.9%
51-60	47	12.2%
61+	24	6.3%
Residence		
Urban	233	60.7%
Rural	151	39.3%

Clinical diagnosis distribution

The most common clinical diagnosis was CSOM, accounting for 46.6% of cases, followed by otitis externa (42.7%). A smaller subset of participants presented with both conditions concurrently (10.7%) (Table 2).

**Table 2:** Frequency and distribution of CSOM, otitis externa, and co-infections by bacterial and fungal pathogens (N=384). CSOM: chronic suppurative otitis media

Variables	Frequency (n)	Percentage (%)
Diagnosis		
CSOM	179	46.6%
Otitis externa	164	42.7%
Both	41	10.7%
Pathogen Type		
Bacterial	192	50.0%
Fungal	110	28.6%
Both	82	21.4%
Specific Bacterial Pathogen		
Staphylococcus aureus	81	21.1%
Pseudomonas aeruginosa	133	34.6%
Streptococcus pneumoniae	79	20.6%
Escherichia coli	91	23.7%
Specific Fungal Pathogen		
Candida spp.	112	29.2%
Aspergillus spp.	191	49.7%
Fusarium spp.	81	21.1%

Pathogen type and frequency

Bacterial infections were the most prevalent, comprising 50.0% of the cases, followed by fungal infections at 28.6%. A smaller proportion of cases involved both bacterial and fungal pathogens (21.4%). The most common bacterial pathogen was Pseudomonas aeruginosa (34.6%), while the most common fungal pathogen was Aspergillus spp. (49.7%) (Table 2).

Association of pathogens with diagnosis

Bacterial infections were most frequently associated with both CSOM and otitis externa, with P. aeruginosa being the predominant pathogen. Fungal infections were primarily caused by Aspergillus spp., followed by Candida spp. The distribution of pathogen types across the different clinical diagnoses (CSOM, otitis externa, and both conditions) was not statistically significant (p = 0.094, chi-square = 7.943) (Table 3).

**Table 3:** Association of bacterial and fungal pathogen and specific pathogen type with CSOM and otitis externa (N=384). Note: P-value <0.05 is considered significant. CSOM: chronic suppurative otitis media

VARIABLES	CSOM (n%)	Otitis Externa (%)	Both (%)
Pathogen Type			
Bacterial	89 (49.7%)	82 (50.0%)	21 (51.2%)
Fungal	51 (29.1%)	41 (25.0%)	17 (41.5%)
Both	38 (21.2%)	41 (25.0%)	3 (7.3%)
P-value (chi-square)	0.094 (7.943)		
Specific Bacterial Pathogen			
Staphylococcus aureus	37 (20.7%)	36 (22.0%)	8 (19.5%)
Pseudomonas aeruginosa	62 (34.6%)	54 (32.9%)	17 (41.5%)
Streptococcus pneumoniae	38 (21.2%)	36 (22.0%)	5 (12.2%)
Escherichia coli	42 (23.5%)	38 (23.2%)	11 (26.8%)
P-value (chi-square)	0.868 (2.592)		
Specific Fungal Pathogen			
Candida spp.	54 (30.2%)	46 (28.0%)	12 (29.3%)
Aspergillus spp.	94 (52.5%)	76 (46.3%)	21 (51.2%)
Fusarium spp.	31 (17.3%)	42 (25.6%)	8 (19.5%)
P-value (chi-square)	0.455 (3.651)		

Specific pathogens and their distribution

Among the bacterial pathogens, P. aeruginosa was most prevalent across all diagnoses, followed by Escherichia coli and Streptococcus pneumoniae. For fungal pathogens, Aspergillus spp. was the most common, with Candida spp. and Fusarium spp. also frequently identified. There were no statistically significant differences in the distribution of specific bacterial pathogens (p = 0.868, chi-square = 2.592) or specific fungal pathogens (p = 0.455, chi-square = 3.651) between CSOM and otitis externa (Table 3).

Mixed infections

Mixed infections were identified in 21.4% of cases, with both bacterial and fungal pathogens isolated from a single specimen. The distribution of mixed infections did not show a significant association with any specific clinical diagnosis. The most common mixed infection pair included P. aeruginosa and Aspergillus spp.

Summary

Overall, bacterial pathogens were more prevalent than fungal pathogens, with P. aeruginosa and Aspergillus spp. being the leading organisms identified across both CSOM and otitis externa. No statistically significant differences were observed in the distribution of pathogens between the different clinical conditions.

## Discussion

This study examined the microbial profile of CSOM and otitis externa in patients with persistent otorrhea in Karachi, Pakistan, a setting where warm, humid conditions and limited healthcare infrastructure contribute to the high burden of ear infections. Few studies in Pakistan have simultaneously assessed bacterial and fungal co-infections in this population, highlighting a critical gap in understanding the spectrum and coexistence of microbial pathogens in otologic infections.

Our study identified bacterial pathogens as the most prevalent causative agents, with P. aeruginosa representing a substantial proportion. This finding aligns with previous research highlighting P. aeruginosa as a significant contributor to CSOM and otitis externa globally, as reported by Chirwa et al. [8], Schaefer et al. [9], and Taoussi [12], as well as locally by Mansoor et al. [16]. This high prevalence can be attributed to P. aeruginosa's ability to thrive in moist and warm environments, such as the middle ear and external auditory canal, which are often predisposed to infection in cases of CSOM and otitis externa [16,18,19]. Its capacity to form biofilms enhances its persistence and resistance to antibiotics, making it a leading cause of chronic infections [20-22]. These characteristics, along with their ubiquity in both hospital and community environments, necessitate tailored antimicrobial strategies. Our findings reinforce regional trends and underscore the need for heightened infection control measures to mitigate its spread.

Fungal pathogens were also prevalent, constituting 28.6% of cases, with Aspergillus spp. (49.7%) as the most commonly identified fungi. This aligns with findings from other tropical and subtropical regions, such as findings by Enoz et al. [15] in Turkey, where fungal infections, particularly Aspergillus and Candida spp., are frequently observed in otitis externa [23]. These high proportions may be due to environmental factors, such as humidity and poor sanitation, in foster fungal growth and the subsequent challenges of managing these infections. Clinically, these infections demand prolonged treatment courses, often weeks to months, to fully eradicate slow-growing hyphal elements from necrotic tissue. In many LMIC settings, limited laboratory infrastructure, reagent shortages, and a shortage of trained mycology personnel make routine fungal culture and speciation impractical, forcing reliance on empirical antifungal therapy. Such an approach risks both overtreatment, which can drive resistance, and undertreatment, which allows deep-seated infections to persist and become refractory. Taken together, these challenges underscore the urgent need to strengthen mycology diagnostic capacity through the adoption of chromogenic or selective media and basic susceptibility methods and to implement antifungal stewardship protocols that ensure timely, targeted therapy and improved patient outcomes.

Our study also highlighted the polymicrobial nature of these infections, with mixed bacterial and fungal pathogens present in 21.4% of cases. This rate is higher than those reported in some studies, such as 17.8% in Ethiopia [7]. This discrepancy may reflect Karachi’s warm, humid climate and high population density, which favor pathogen growth, as well as healthcare challenges such as limited infection control measures, overcrowded facilities, and a high prevalence of immunocompromised individuals due to malnutrition and diabetes [24,25], and may contribute to the higher frequency of co-infections. Differences in laboratory diagnostic methods, such as the use of more comprehensive pathogen detection panels or more sensitive techniques, may also explain the higher detection rate of mixed infections in our study. Moreover, our use of more comprehensive culture panels and sensitive molecular assays likely increased the detection of co-infections. Clinically, polymicrobial infections carry a greater risk of treatment failure and the emergence of antimicrobial resistance, since mixed pathogens can interact synergistically and complicate therapeutic choices. These findings underscore the need to expand diagnostic capabilities by incorporating advanced culture-based techniques alongside molecular diagnostics and to strengthen infection control and antimicrobial stewardship programs that specifically address mixed bacterial/fungal infections. Finally, given the rising incidence of resistance globally and locally, local empirical treatment guidelines should integrate combined antibacterial and antifungal susceptibility testing to guide more effective, targeted therapies.

Interestingly, pathogen distribution did not differ significantly between CSOM and otitis externa (p = 0.094), suggesting overlapping etiological factors in both conditions [14,26]. Clinically, this implies that empiric antimicrobial regimens, whether antibacterial, antifungal, or a combination, can be selected using a unified approach for both diagnoses in comparable high-risk settings. These findings suggest that empiric treatment approaches may be similar for both conditions in environments characterized by warm, humid climates, poor sanitation, and limited healthcare access, thereby simplifying clinical decision making and optimizing resource use in resource-limited urban centers. Additionally, the urban predominance of participants (60.7%) further emphasizes the role of environmental and socio-economic factors in the disease burden.

The results also highlight the necessity of targeted microbial profiling to optimize treatment protocols. The observed frequency of mixed infections underscores the importance of antifungal susceptibility testing alongside bacterial cultures, particularly in cases refractory to standard antibiotic regimens. Currently, antifungal susceptibility testing is not routinely performed in many LMICs due to cost and technical constraints, which limit the ability to tailor therapy. Our findings advocate for the inclusion of such testing in standard diagnostic protocols. A dual approach, such as a diagnostic and therapeutic approach, meaning concurrent testing and co-administration of antibacterial and antifungal agents when warranted, may improve clinical outcomes, especially in patients unresponsive to empirical antibacterial treatment alone.

Our study has several limitations that warrant consideration. First, the use of a cross-sectional design precludes assessment of temporal or causal relationships between environmental, demographic, and clinical factors and the occurrence of infections. Second, convenient sampling and restriction to two urban hospitals may limit the generalizability of the findings to broader populations, particularly rural communities where pathogen prevalence and healthcare access may differ significantly. Third, the exclusion of patients receiving antibiotic therapy, while reducing confounding in pathogen identification, may have led to underrepresentation of resistant organisms or altered pathogen distribution patterns, which might have led to some bias. Furthermore, while the study identified bacterial and fungal pathogens, anti-bacterial/fungal susceptibility testing was not performed, due to resource limitations and the primary focus of the study being pathogen identification rather than treatment outcomes, which limits clinical application in guiding antifungal therapy, especially in cases refractory to standard treatment, as well limits conclusion on resistance patterns. Future longitudinal and intervention studies incorporating randomized sampling across diverse geographic regions and including antifungal resistance profiling are recommended to enhance the external validity and clinical relevance of these findings. This study also did not collect data on symptom duration, volume of otorrhea, or hearing loss, which may have provided valuable insights into the correlation between clinical severity or chronicity and pathogen type.

Our findings provide updated insights into the microbial landscape of chronic otorrhea in a resource-limited urban setting. The high burden of bacterial, fungal, and polymicrobial infections calls for improved diagnostic infrastructure, routine susceptibility testing, and integrated treatment strategies. These insights may inform infection control and empiric treatment guidelines in similar urban, resource-constrained regions.

## Conclusions

In conclusion, our study demonstrates that P. aeruginosa and Aspergillus spp. are the predominant bacterial and fungal pathogens in CSOM and otitis externa in Karachi, Pakistan, highlighting the necessity of routine microbial profiling in clinical settings to inform evidence-based, localized treatment protocols. The role of environmental factors, such as poor sanitation and a humid climate, in driving disease burden underscores the importance of comprehensive diagnostics, including antifungal susceptibility testing, to effectively manage polymicrobial infections. While the cross-sectional design and absence of resistance profiling limit causal inference, these findings lay the groundwork for future longitudinal and interventional studies aimed at refining local treatment guidelines and improving patient outcomes in resource-constrained settings.

## Appendices

**Table 4:** Evaluation form.

Section 1: Demographic Information
Participant ID:
Do you consent to participate in this study?
Yes
No
Age:
11-20 years
21-30 years
31-40 years
41-50 years
51-60 years
61 years and above
Sex:
Male
Female
Other:_______________________
Occupation: _______________________
Residence:
Urban
Rural
Section 2: Diagnosis
Diagnosis:
Chronic Suppurative Otitis Media (CSOM)
Otitis Externa
Both
Section 3: Pathogen Characterization
Pathogen Type (Select one):
Bacterial
Fungal
Both
If Bacterial Pathogen is Identified, Specify:
Staphylococcus aureus
Pseudomonas aeruginosa
Streptococcus pneumoniae
Escherichia coli
Other: ______________________
If Fungal Pathogen is Identified, Specify:
Candida spp.
Aspergillus spp.
Fusarium spp.
Other: ______________________
